# New Oral Anticoagulants vs. Vitamin K Antagonists Among Patients With Cardiac Amyloidosis: Prognostic Impact

**DOI:** 10.3389/fcvm.2021.742428

**Published:** 2021-11-30

**Authors:** Eve Cariou, Kevin Sanchis, Khailène Rguez, Virginie Blanchard, Stephanie Cazalbou, Pauline Fournier, Antoine Huart, Murielle Roussel, Pascal Cintas, Michel Galinier, Didier Carrié, Philippe Maury, Yoan Lavie-Badie, Olivier Lairez

**Affiliations:** ^1^Department of Cardiology, Rangueil University Hospital, Toulouse, France; ^2^Cardiac Imaging Center, Toulouse University Hospital, Toulouse, France; ^3^Department of Nuclear Medicine, Toulouse University Hospital, Toulouse, France; ^4^Medical School, Toulouse III Paul Sabatier University, Toulouse, France; ^5^Department of Nephrology and Referral Center for Rare Diseases, Rangueil University Hospital, Toulouse, France; ^6^Department of Hematology, IUC Oncopole, Toulouse, France; ^7^Department of Neurology, Purpan University Hospital, Toulouse, France

**Keywords:** cardiac amyloidosis, direct oral anticoagulants, vitamin K-antagonists (VKAs), prognosis, atrial arrhythmia

## Abstract

**Background:** Atrial arrhythmia (AA) is common among patients with cardiac amyloidosis (CA), who have an increased risk of intracardiac thrombus. The aim of this study was to explore the prognostic impact of vitamin K-antagonists (VKA) and direct oral anticoagulants (DOAC) in patients with CA.

**Methods and Results:** 273 patients with CA and history of AA with long term anticoagulation−69 (25%) light chain amyloidosis (AL), 179 (66%) wild-type transthyretin amyloidosis (ATTRwt) and 25 (9%) variant transthyretin amyloidosis (ATTRv)–were retrospectively included between January 2012 and July 2020. 147 (54%) and 126 (46%) patients received VKA and DOAC, respectively. Patient receiving VKA were more likely to have AL with renal dysfunction, higher NT-proBNP and troponin levels. Patients with ATTRwt were more likely to receive DOAC therapy. There were more bleeding complications among patients with VKA (20 versus 10%; *P* = 0.013) but no difference for stroke events (4 vs. 2%; *P* = 0.223), as compared to patients with DOAC. A total of 124 (45%) patients met the primary endpoint of all-cause mortality: 96 (65%) and 28 (22%) among patients with VKAs and DOACs, respectively (*P* < 0.001). After multivariate analysis including age and renal function, VKA was no longer associated with all-cause mortality.

**Conclusion:** Among patients with CA and history of AA receiving oral anticoagulant, DOACs appear to be at least as effective and safe as VKAs.

## Introduction

Atrial arrhythmia (AA) is common in patients with cardiac amyloidosis (CA). AA is more frequent in wild-type transthyretin amyloidosis (ATTRwt) than light chain amyloidosis (AL) or variant transthyretin amyloidosis (ATTRv). Patients with CA have an increased risk of intracardiac thrombus, reaching 33% in autopsy series (1), and its more frequent in AL with more fatal embolic events despite normal sinus rhythm and preserved ejection fraction (2). This high prevalence of intracardiac thrombus justify to recommend systematic transesophageal echocardiography before direct-current cardioversion despite correct anticoagulation with international normalized ratio between 2 and 3 more than 3 weeks (3). Furthermore, the CHA2DS2-VASc score is not reliable in cardiac amyloidosis since 67% of patients with thrombus present in pre-cardioversion transesophageal echocardiography had a score of 1 (4). Vitamin K-antagonists (VKAs) were the first anticoagulants used in patients with history of AA requiring anticoagulation, but direct oral anticoagulants (DOACs) have been introduced in previous and current European and US guidelines for the management of atrial fibrillation as suitable alternatives to VKAs for stroke prevention in AA (3, 5). However, efficiency and safety of traditional anticoagulants vs. DOACs in patients with CA have never been explored.

The aim of this study was to explore the prognostic impact of VKAs and DOACs in patients with CA requiring anticoagulation.

## Methods

### Study Population

**Figure 1:**
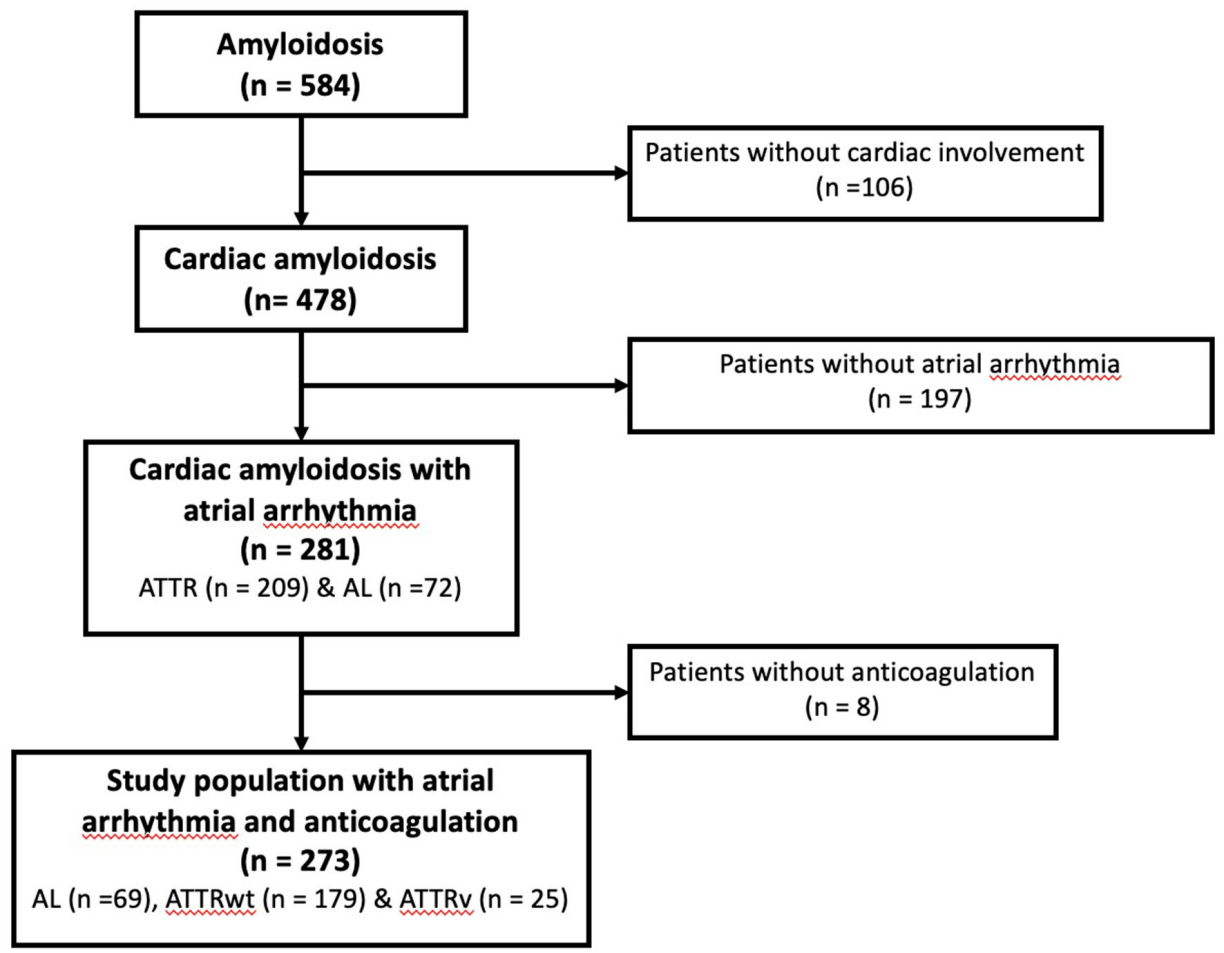
Study flowchart for the patient selection.

Between January 2012 and July 2020, all consecutive patients with CA diagnosed at the University Hospital of Toulouse, France, were retrospectively enrolled in this cohort. Details of the population selection have previously been described (6) and the flow chart for the population study selection is presented in Figure 1. Briefly, only patients with history of AA receiving oral anticoagulant were included in the final analysis. Patient's medical records were reviewed to collect anticoagulant treatments during management and at the time of follow-up.

AA were defined by sustained atrial fibrillation, common atrial flutter or atrial tachycardia. Oral anticoagulant drugs were represented either by VKA (Coumadine, Fluindione or Acecoumarol) or DOAC (Dabigatran, Rivaroxaban or Apixaban).

The investigation conforms with the principles outlined in the Declaration of Helsinki. All patients were informed at the admission that their clinical data could be used for research purpose and gave their consent. The study was approved by the French Data Protection Authority (*Commission Nationale de l'Informatique et des Libertés*, #2205212v0).

### Clinical Endpoint and Follow-Up

Follow-up was assessed by electronic chart review or by phone interview of patient's general practitioner/cardiologist, patient or family for the clinical endpoint of all-cause and cardiovascular mortality. The secondary outcomes were embolic events (essentially stroke), left atrial appendage thrombus and bleeding event (anticoagulation complication). Major bleeding event was defined by symptomatic bleeding or bleeding causing a fall in hemoglobin level of 2g/dL according to International Society on Thrombosis and Haemostasis bleeding scale (7) and minor bleeding event was defined by all non-major bleedings. Patients without contact up to 6 months were considered as lost to follow-up. Only patients with available demographic data were used for survival analysis.

### Statistical Analysis

Continuous variables were tested for normal distribution using the Kolmogorov–Smirnov test and expressed as mean ± standard deviation. Values not normally distributed were presented as medians with interquartile ranges (IQR). Nominal values were expressed as numbers and percentages. Comparisons of numerical variables was compared using the Student's test or the Mann-Whitney rank sum test when appropriate. Nominal variables were investigated by the χ2 test or the Fisher exact as suitable. Univariable and multivariable Cox proportional-hazards regression analysis were performed to test the association of variables associated with all-cause mortality. Univariate variables with a *P*-value of <0.05 were entered into the multivariate logistic models. All-cause mortality was then summarized using Kaplan-Meier survival curve and log rank test was used for initial comparisons. Patients were censored at the time of death. Differences were considered statistically significant for *P*-values of <0.05. All analyses were performed using standard statistical software SPSS version 20 (SPSS Inc., Chicago, Illinois).

## Results

### Baseline Characteristics

Among the 478 patients with CA and complete medical records initially screened, 273 (57%) had a previous history of AA with long-term anticoagulation during the follow-up: 69 (25%) AL, 179 (66%) ATTRwt and 25 (9%) ATTRv (Figure 1).

There were 147 (54%) and 126 (46%) patients receiving VKA and DOAC, respectively. Eigthy-one (55%), 67 (46%) and 2 (1%) received Warfarin, Fluindione and Acenocoumarol, respectively among patients with VKA; and 77 (61%), 35 (28%) and 14 (11%) received Apixaban, Rivaroxaban and Dabigatran, respectively among patients with DOAC. Among patients with DOAC, 55 (44%) received a low dose in accordance with the recommendations for adapting doses according to their age, weight or renal function. The baseline clinical characteristics of the study population are summarized in Table 1. There were more AL patients, renal dysfunction, higher NT-proBNP and troponin levels and associated antiplatelet therapy among patient receiving VKA. Patients with wtATTR were more likely to receive DOAC. Among patients treated with VKA treatment, 66 (45%) reported an under target International Normalized Ratio during the follow-up period.

**Table 1:** Population demographics.

	**Whole**	**VKAs**	**DOACs**	***P*-value**
	***n* = 273**	***n* = 147**	***n* = 126**	
Age at diagnosis, years	78 ± 10	77 ± 11	79 ± 8	0.129
Male, *n* (%)	211 (77)	107 (73)	104 (83)	0.055
Body mass index, kg/m2	25 ± 4	25 ± 4	25 ± 4	0.339
Diabetes mellitus, *n* (%)	40 (15)	24 (16)	16 (13)	0.385
Vascular disease, *n* (%)	62 (23)	31 (21)	31 (25)	0.529
Hypertension, *n* (%)	127 (47)	69 (47)	58 (46)	0.840
Amyloidosis				
AL	69 (25)	54 (37)	15 (12)	<0.001
ATTRwt	179 (66)	85 (58)	94 (75)	0.004
ATTRv	25 (9)	8 (5)	17 (13)	0.022
Atrial arrhythmia				
Atrial fibrillation, *n* (%)	267 (98)	145 (99)	122 (97)	0.308
Flutter, *n* (%)	46 (17)	24 (16)	22 (17)	0.803
Embolic event, *n* (%)	52 (19)	31 (21)	21 (17)	0.354
CHA2DS2-VASc score	4 ± 1	4 ± 1	4 ± 1	0.688
NYHA stage, *n* (%)				
I	48 (18)	23 (16)	25 (20)	0.375
II	110 (40)	59 (40)	51 (40)	0.942
III	87 (32)	44 (30)	43 (34)	0.477
IV	23 (8)	19 (13)	4 (3)	0.004
Biology				
Creatinine, μmol/l	152 ± 99	183 ± 122	114 ± 33	<0.001
Glomerular filtration rate, ml/min	46 ± 21	39 ± 22	55 ± 18	<0.001
NT pro-BNP, ng/ml	4,269 [2,315–9,053]	5,239 [2,821–15,793]	3,415 [1,813–5,810]	<0.001
Troponin, ng/ml	84 [53–136]	111 [71–164]	71 [48–106]	<0.001
Echocardiography				
Left ventricular ejection fraction, %	49 ± 12	46 ± 12	51 ± 12	0.002
Global longitudinal strain, %	11 ± 4	10 ± 4	13 ± 3	<0.001
Left atrial volume index, ml/m2	55 ± 16	56 ± 17	53 ± 15	0.391
Deceleration time, ms	186 ± 72	183 ± 84	189 ± 57	0.112
E/Ea lateral	16 ± 6	16 ± 6	15 ± 7	0.207
Medications				
Digoxin, *n* (%)	7 (3)	17 (12)	2 (2)	0.340
Beta-blocker, *n* (%)	60 (22)	43 (29)	17 (13)	0.002
Amiodarone, *n* (%)	144 (53)	87 (59)	57 (45)	0.058
Antiplatelet, *n* (%)	59 (22)	39 (27)	20 (16)	0.033
Switch anticoagulation, *n* (%)	24 (9)	13 (9)	11 (9)	0.974
Complications				
Sludge or thrombus, *n* (%)	26 (10)	19 (13)	7 (6)	0.539
Anticoagulation complication, *n* (%)	42 (15)	30 (20)	13 (10)	0.013
Minor bleeding, *n* (%)	22 (8)	13 (9)	10 (8)	0.099
Major bleeding, *n* (%)	20 (7)	17 (12)	3 (2)	<0.001
Stroke, *n* (%)	8 (3)	6 (4)	2(2)	0.223

*DOACs, direct oral anticoagulants; VKAs, vitamin K antagonists*.

### Impact of Anticoagulant Therapy on Outcomes

There were more bleeding complications among patients with VKA as compared to patients with DOAC, with 30 (20%) vs. 13 (10%) events, respectively, *P* = 0.013; but no difference for stroke, with 6 (4%) vs. 2 (2%) events, respectively, *P* = 0.223. Thirty-six patients presented a stroke before the introduction of curative oral anticoagulation.

**Figure 2:**
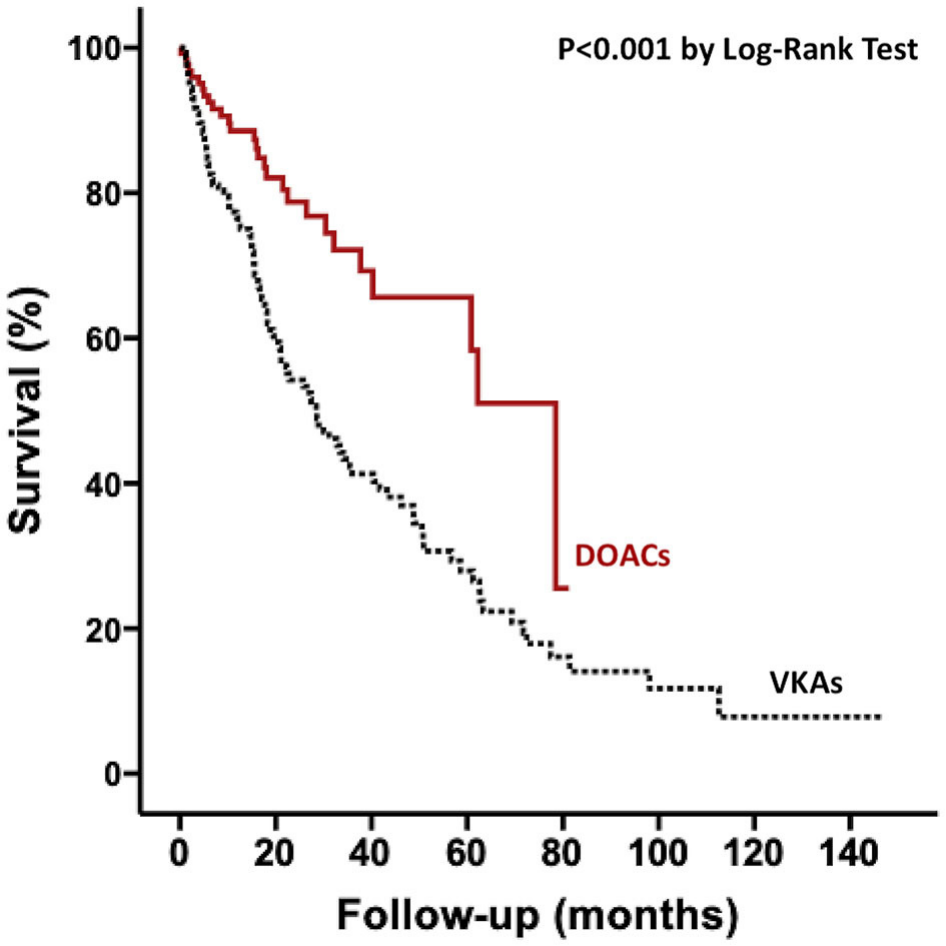
Kaplan–Meier curves for all-cause mortality according to anticoagulation treatment for the whole population. DOACs, direct oral anticoagulants; VKAs, vitamin K antagonists.

Survival data were available for all patients, with a median follow-up of 18 months with no significant difference between patients on VKA and DOAC respectively (18 [IQR 8-43] and 18 [IQR 8-32], *p* = 0.286) A total of 124 (45%) patients met the primary endpoint of all-cause mortality: 96 (65%) and 28 (22%) among patients with VKA and DOAC, respectively (*P* < 0.001). In univariate analysis, there was an over-risk of all-cause mortality associated with VKA treatment compared DOAC treatment (Figure 2). In multivariate analysis, age, NYHA 3 and 4 class, NT-proBNP level, renal dysfunction, global longitudinal strain level, major bleeding and beta-blocking drugs were still associated with an increased risk of mortality (Table 2). However, type of oral anticoagulation (VKA vs. DOAC) was no more linked to survival after adjustment of these variables.

**Table 2:** Cox regression analysis for the occurrence of all-cause mortality in the whole population.

	**Univariate**		**Multivariate**	
	**HR (95% CI)**	***P*-value**	**HR (95% CI)**	***P*-value**
Age, per year	1.02 (1.00–1.04)	0.031	0.86 (0.78–0.95)	0.002
Male gender	1.11 (0.71–1.74)	0.635		
Body mass index, per unit	0.97 0.92–1.02)	0.228		
Diabetes mellitus	1.78 (1.13–2.79)	0.012	3.83 (0.42–34.6)	0.232
Vascular disease	1.10 (0.72–1.68)	0.653		
Hypertension	1.06 (0.75–1.52)	0.731		
Amyloidosis: AL vs. ATTRwt	0.99 (0.69–1.45)	0.997		
Atrial fibrillation				
Permanent vs. non-permanent	1.21 (0.85–1.72)	0.297		
CHA2DS2-VASc score, per unit	1.07 (0.95–1.20)	0.251		
Embolic vs. non-embolic event	1.13 (0.72–1.77)	0.594		
NYHA stage III & IV vs. I & II	1.66 (1.18–2.33)	0.003	15.4 (2.2–109)	0.006
Biology				
Glomerular filtration rate, per ml/min	0.99 (0.98–0.99)	0.002	0.89 (0.81–0.97)	0.008
NT pro-BNP, per mg/ml	1.00 (1.00–1.00)	<0.001	1.00 (0.98–1.02)	0.015
Troponin, per 10 ng/ml	1.00 (1.00–1.00)	0.018	1.01 (1.00–1.02)	0.614
Echocardiography				
Left ventricular ejection fraction, per %	0.99 (0.99–1.00)	0.045	1.04 (0.97–1.12)	0.244
Global longitudinal strain, per %	0.92 (0.81–1.04)	<0.001	0.71 (0.55–0.92)	0.008
Left atrial volume index, per ml/m^2^	1.01 (0.99–1.03)	0.154		
Deceleration time, per ms	0.99 (0.99–1.00)	0.482		
E/Ea lateral	1.04 (1.00–1.07)	0.027	1.02 (0.9–1.15)	0.804
Medications				
VKA vs. DOAC	2.36 (1.54–3.61)	<0.001	2.29 (0.53–10)	0.269
Digoxin vs. no digoxin	1.00 (0.32–3.14)	0.996		
Beta-blocker vs. no beta-blockers	1.53 (1.02–2.31)	0.041	25 (1.7–357)	0.018
Amiodarone vs. no amiodarone	0.96 (0.67–1.38)	0.809		
Antiplatelet vs. no antiplatelet	1.27 (0.84–1.91)	0.256		
Complications				
Stroke	1.19 (0.38–3.76)	0.768		
Sludge or thrombus	1.34 (0.71–2.53)	0.361		
Anticoagulation complication	1.59 (1.05–2.42)	0.030	0.53 (0.06–4.93)	0.574
Minor bleeding	1.26 (0.68–2.37)	0.464		
Major bleeding	2.10 (1.14–3.86)	0.018	79 (4.8–1299)	0.002

*DOACs, direct oral anticoagulants; VKAs, vitamin K antagonists*.

### Sub-Population of Patients With ATTRwt

Baseline characteristics of patients with ATTRwt are presented in annex. Patients receiving VKA were more symptomatic, were more likely to have renal dysfunction, lower ejection fraction and lower global longitudinal strain and to receive beta-blocker and antiplatelet therapy.

In the univariate analysis, there was an associated increased risk of VKA treatment over DOAC treatment for the composite endpoint of stroke and bleeding complications (HR 2,76 [1,28–5,97], *p* = 0.010) that disappeared after multivariate analysis (HR 1,84 [0,77–4,43] *p* = 0.171).

**Figure 3:**
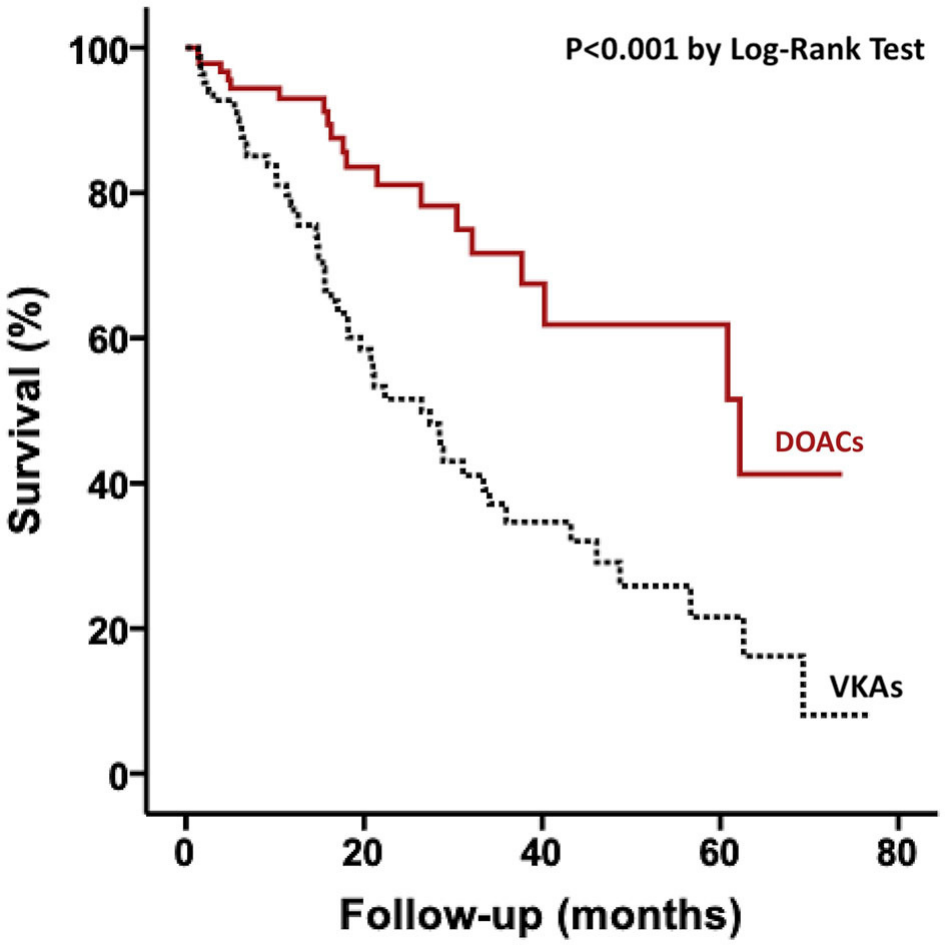
Kaplan–Meier curves for all-cause mortality according to anticoagulation treatment among patients with ATTRwt. DOACs, direct oral anticoagulants; VKAs, vitamin K antagonists.

Regarding outcomes, patients receiving VKA had more major bleeding during the follow-up as compared to patients receiving DOAC (14 vs. 2%, respectively; *p* < 0.001) but there was no significant difference for stroke events (7 vs. 2%, respectively; *p* = 0.074). In univariate analysis, there was an over-risk of all-cause mortality associated with VKA treatment compared DOAC treatment (Figure 3). After multivariate analysis, only glomerular filtration rate was associated with an increased risk of mortality (Table 3), but type of oral anticoagulation therapy was no more linked with all-cause mortality.

**Table 3:** Cox regression analysis for the occurrence of all-cause mortality among patients with ATTRwt.

	**Univariate**		**Multivariate**	
	**HR (95% CI)**	***P*-value**	**HR (95% CI)**	***P*-value**
Age, per year	1.07 (1.02–1.13)	0.004	0.98 (0.86–1.12)	0.817
Male gender	1.96 (0.79–4.90)	0.149		
Body mass index, per unit	0.94 (0.87–1.00)	0.065		
Diabetes mellitus	1.63 (0.91–2.89)	0.098		
Vascular disease	1.19 (0.69–2.05)	0.523		
Hypertension	1.23 (0.76–1.99)	0.408		
Atrial fibrillation				
Permanent vs. non-permanent	1.04 (0.64–1.69)	0.877		
CHA2DS2-VASc score, per unit	1.08 (0.91–1.28)	0.366		
Embolic vs. non-embolic event	0.86 (0.49–1.54)	0.617		
NYHA stage III & IV vs. I & II	1.79 (1.10–2.91)	0.019	1.86 (0.41–8.55)	0.425
Biology				
Glomerular filtration rate, per ml/min	0.98 (0.96–0.99)	0.001	0.88 (0.80–0.97)	0.008
NT pro-BNP, per mg/ml	1.00 (1.00–1.00)	<0.001	1.00 (1.00–1.00)	0.448
Troponin, per 10 ng/ml	1.00 (0.99–1.00)	0.363		
Echocardiography				
Left ventricular ejection fraction, per %	0.99 (0.97–1.01)	0.226		
Global longitudinal strain, per %	0.91 (0.83–0.99)	0.027	0.90 (0.72–1.12)	0.345
Left atrial volume index, per ml/m^2^	1.00 (0.98–1.03)	0.779		
Deceleration time, per ms	1.00 (0.99–1.01)	0.672		
E/Ea lateral	1.02 (0.97–1.07)	0.421		
Medications				
VKA vs. DOAC	2.77 (1.63–4.70)	<0.001	1.09 (0.26–4.60)	0.910
Digoxin vs. no digoxin	0.05 (0–15000)	0.639		
Beta-blocker vs. no beta-blockers	1.41 (0.82–2.42)	0.218		
Amiodarone vs. no amiodarone	0.69 (0.42–1.14)	0.150		
Antiplatelet vs. no antiplatelet	1.09 (0.62–1.92)	0.760		
Complications				
Stroke	1.06 (0.59 – 1.92)	0.850		
Sludge or thrombus	0.98 (0.42–2.25)	0.953		
Anticoagulation complication	1.77 (1.00–3.13)	0.050		
Minor bleeding	1.19 (0.49–2.92)	0.704		
Major bleeding	3.22 (1.47–7.07)	0.004	2.67 (0.39–18)	0.314

*DOACs, direct oral anticoagulants; VKAs, vitamin K antagonists*.

### Sub-Population of Patients With AL

**Figure 4:**
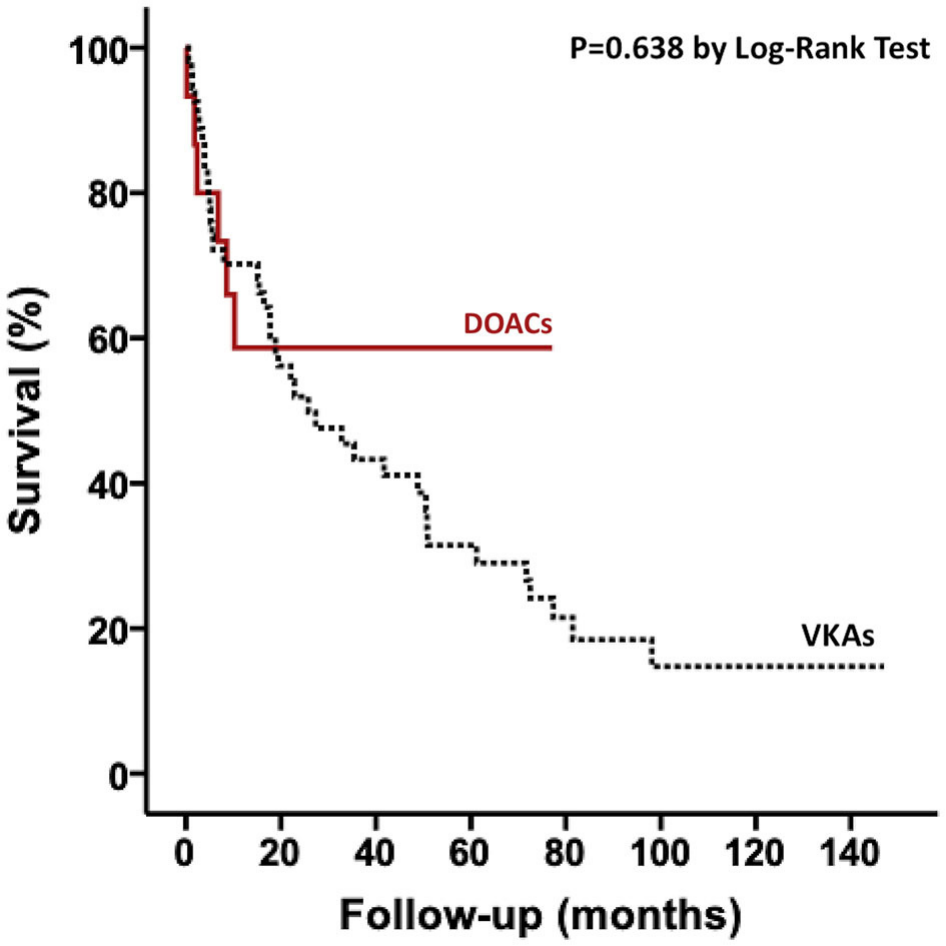
Kaplan–Meier curves for all-cause mortality according to anticoagulation treatment among patients with AL. DOACs, direct oral anticoagulants; VKAs, vitamin K antagonists.

Baseline characteristics of patients with AL are presented in annex. There was no difference between patients receiving VKA and DOAC except for higher proportion of renal dysfunction among patients receiving VKA. Regarding outcomes, there was no difference between groups for bleeding complications (22 vs. 13% for VKA and DOAC, respectively; *P* = 0.449) and there was not any stroke event over the follow-up. There was no association between type of anticoagulation therapy and all-cause mortality (Figure 4). After multivariate analysis, only E/Ea ratio on transthoracic echocardiography was associated with increased risk of mortality (Table 4).

**Table 4:** Cox regression analysis for the occurrence of all-cause mortality among patients with AL.

	**Univariate**		**Multivariate**	
	**HR (95% CI)**	***P*-value**	**HR (95% CI)**	***P*-value**
Age, per year	1.03 (0.99–1.06)	0.082		
Male gender	1.09 (0.59–1.99)	0.790		
Body mass index, per unit	1.00 (0.93–1.08)	0.942		
Diabetes mellitus	1.87 (0.89–3.92)	0.095		
Vascular disease	1.70 (0.81–3.54)	0.154		
Hypertension	1.03 (0.58–1.86)	0.898		
Atrial fibrillation				
Permanent vs. non-permanent	1.52 (0.85–2.71)	0.158		
CHA2DS2-VASc score, per unit	1.12 (0.91–1.36)	0.264		
Embolic vs. non-embolic event	1.93 (0.91–4.08)	0.084		
NYHA stage III & IV vs. I & II	1.62 (0.90–2.90)	0.105		
Biology				
Glomerular filtration rate, per ml/min	0.99 (0.98–1.01)	0.744		
NT pro-BNP, per mg/ml	1.00 (1.00–1.00)	0.018	1.00 (1.00–1.00)	0.078
Troponin, per 10 ng/ml	1.00 (1.00–1.00)	0.056		
Echocardiography				
Left ventricular ejection fraction, per %	0.98 (0.96–1.01)	0.391		
Global longitudinal strain, per %	0.87 (0.77–0.98)	0.017	1.01 (0.84–1.21)	0.884
Left atrial volume index, per ml/m^2^	1.01 (0.99–1.03)	0.120		
Deceleration time, per ms	0.99 (0.98–0.99)	0.027	1.00 (0.99–1.01)	0.969
E/Ea lateral	1.06 (1.00–1.12)	0.025	1.14 (1.03–1.27)	0.012
Medications				
VKA vs. DOAC	1.23 (0.51–2.94)	0.639		
Digoxin vs. no digoxin	1.11 (0.34–3.60)	0.858		
Beta-blocker vs. no beta-blockers	1.51 (0.76–2.98)	0.236		
Amiodarone vs. no amiodarone	1.07 (0.58–1.95)	0.821		
Antiplatelet vs. no antiplatelet	1.56 (0.80–3.04)	0.185		
Complications				
Sludge or thrombus	1.29 (0.41–4.01)	0.657		
Anticoagulation complication	1.40 (0.70–2.78)	0.331		
Minor bleeding	1.24 (0.47–3.26)	0.661		
Major bleeding	1.28 (0.41–3.97)	0.665		

*DOACs, direct oral anticoagulants; VKAs, vitamin K antagonists*.

## Discussion

This retrospective study on 273 patients with cardiac amyloidosis and a history of AA requiring anticoagulation shows that DOACs can be used safely without increasing mortality, embolic events or bleeding complications, which were lower compared to VKA therapy. Conversely, our study shows that there appears to be an over risk of mortality under anticoagulant treatment with VKA, which disappears after adjustment for age and renal function, regardless of the subtype of amyloidosis.

Indeed, we can see that patients with renal insufficiency were more often on VKAs, which can be explained by a reluctance of physicians to prescribe DOACs for these patients, especially at the beginning of the census of our cohort, which coincides with the beginning of the marketing authorization of DOACs.

To our knowledge, this is the first study exploring the impact of the type of anticoagulant on all-cause mortality in a population of CA. The ARISTOTLE trial (8) was the only one to show a decrease in mortality with apixaban as compared to warfarin in patients with atrial fibrillation or atrial flutter but without specific mention of CA. The recent study by Mitrani and co-authors did not find difference between patients with CA received VKAs and DOACs regarding thrombotic events and major bleeds after a 2.4-year follow-up (9). However, they did not report the impact of the type of anticoagulant on all-cause mortality. To date, the use of DOACs in patients with cardiac amyloidosis and supraventricular tachycardia was minor in the published series (10, 11). No cohort study has reported a difference in prognosis according to the type of anticoagulant used. Our study shows that despite an apparent over-risk of VKAs, there is no difference in mortality between anticoagulants and DOACs can be used safely.

In contrast to the Mitrani study (9), our cohort showed a difference in terms of bleeding complications between groups of anticoagulants, which were more frequent among patients receiving VKA. This could be explained by the fact that patients receiving VKA had more impaired renal function and therefore a higher HAS-BLED hemorrhagic score, which would need to be proven by a multivariate analysis regarding bleeding complications (which could not be done due to a lack of events).

Furthermore, the high rate of bleeding complications in this population of CA must be noted and integrated into the benefit-risk balance of such a therapy. Indeed, we know that these are fragile patients with disturbed hemostasis and impaired renal function, which may explain such a high rate of hemorrhagic complications in comparison with the usual population of patients with atrial fibrillation.

Cardiac amyloidosis has been shown to expose to an over-risk of embolic events with an increased prevalence of atrial thrombus at transesophageal echocardiography (12) or surgery (13). However, despite this increased risk confirmed by our study, there is no difference in events between patients treated with VKA and patients treated with DOACs.

These results must be interpreted with caution, as the patient profile is not the same and the excess mortality of patients on VKAs could simply be explained by a selection bias induced by age and renal function that contraindicates the use of DOACs. Indeed, renal function has clearly been shown to be a major prognostic marker in patients with CA, particularly wtATTR (14).

## Limitations

This study shares all the limitations and bias associated with a retrospective and single-center study. Although the number of deaths is sufficient to conclude on all-cause mortality, the number of thrombo-embolic and hemorrhagic events remains too small to draw definitive conclusions. Ultimately, true safety of DOACs will only be demonstrated by randomized study.

## Conclusion

The choice of therapeutics to be used in CA differs in many ways from other heart failure patients. The thrombotic profile of these patients often requires the use of anticoagulation, the benefit of which may be offset by an increased risk of hemorrhage. The choice between VKA and DOACs can therefore sometimes be difficult. Our study shows that DAOCs can be used without an over-risk of event compared to the standard VKA treatment widely used in clinical practice.

## On Behalf of the Toulouse Amyloidosis Research Network Collaborators

**Blandine Acket**, Department of Neurology, Purpan University Hospital, Toulouse, France; **Laurent Alric**, Department of Internal Medicine and Digestive Diseases, Purpan University Hospital, Toulouse, France; **Christophe Bureau**, Department of Hepatology-Gastroenterology, Rangueil University Hospital, Toulouse, France; **Dominique Chauveau**, Department of Nephrology and Referral Center for Rare Diseases, Rangueil University Hospital, Toulouse, France; **Magali Colombat**, Department of Pathology, IUCT Oncopôle, Toulouse, France; **Audrey Delas**, Department of Pathology, IUCT Oncopôle, Toulouse, France; **Delphine Dupin-Deguine**, Department of Genetic, Toulouse University Hospital, Toulouse, France; **Stanislas Faguer**, Department of Nephrology and Referral Center for Rare Diseases, Rangueil University Hospital, Toulouse, France; **Bénédicte Puissant**, Immunology Laboratory, Toulouse University Hospital, Toulouse, France; **Grégory Pugnet**, Department of Internal Medicine and Digestive Diseases, Purpan University Hospital, Toulouse, France; **Grégoire Prévot**, Department of Pneumology, Toulouse University Hospital, Toulouse, France; **David Ribes**, Department of Nephrology and Referral Center for Rare Diseases, Rangueil University Hospital, Toulouse, France; **Laurent Sailler**, Department of Internal Medicine, Toulouse University Hospital, Toulouse, France.

## Data Availability Statement

The raw data supporting the conclusions of this article will be made available by the authors, without undue reservation.

## Author Contributions

OL designed and drove the study. EC analyzed and interpreted the clinical data and wrote the manuscript. KS and KR collected the clinical data. SC, VB, and PF collected the clinical events and the prognosis. YL-B performed the statistical analysis. AH, PC, MR, MG, and DC contributed to the discussion and the reviewing. PM led the study, revised the manuscript, and gave final approval of the version to be published. All authors contributed to the article and approved the submitted version.

## Conflict of Interest

The authors declare that the research was conducted in the absence of any commercial or financial relationships that could be construed as a potential conflict of interest.

## Publisher's Note

All claims expressed in this article are solely those of the authors and do not necessarily represent those of their affiliated organizations, or those of the publisher, the editors and the reviewers. Any product that may be evaluated in this article, or claim that may be made by its manufacturer, is not guaranteed or endorsed by the publisher.

## Abbreviations

AA: Atrial arrhythmia
AL: light chain amyloidosis
ATTR: transthyretin amyloidosis
ATTRv: variant transthyretin amyloidosis
ATTRwt: wild-type transthyretin amyloidosis
CA: cardiac amyloidosis
DOAC: direct oral anticoagulant
VKA: vitamin K-antagonist.
