# Supplementary material for: New Oral Anticoagulants vs. Vitamin K Antagonists Among Patients With Cardiac Amyloidosis: Prognostic Impact
Source: Front Cardiovasc Med. 2021 Nov 30;8:742428. doi: 10.3389/fcvm.2021.742428 (PMC8670403; doi:10.3389/fcvm.2021.742428)
Supplement: Supplementary file 1 [file Table_1.DOCX]

**SUPPLEMENTS**

## Demographics of ATTRwt population

|  |  | **ATTRwt** | **VKAs** | **DOACs** | **P-value** |
| --- | --- | --- | --- | --- | --- |
|  |  | **n= 179** | **n=85** | **n=94** |  |
| Age at diagnosis, years | | 83±6 | 83±5 | 82±6 | 0.259 |
| Male, n (%) | | 151(84) | 70 (82) | 81 (86) | 0.423 |
| Body mass index, kg/m2 | | 25±4 | 25±4 | 25±4 | 0.786 |
| Diabetes mellitus, n (%) | | 28 (16) | 15 (18) | 13 (14) | 0.461 |
| Vascular disease, n (%) | | 48 (27) | 22 (26) | 26 (28) | 0.825 |
| Hypertension, n (%) | | 91 (51) | 46 (85) | 45 (48) | 0.359 |
| Atrial fibrillation | |  |  |  |  |
|  | Permanent | 113 (63) | 53 (62) | 60 (64) | 0.838 |
|  | CHA2DS2-VASc score | 4±1 | 4.2±1.2 | 3.8±1.4 | **0.026** |
|  | Emboligene | 39 (22) | 23 (27) | 16 (17) | 0.104 |
| NYHA stage, n (%) | |  |  |  |  |
|  | I | 32 (18) | 13 (15) | 19 (20) | 0.413 |
|  | II | 72 (40) | 37 (44) | 35 (37) | 0.380 |
|  | III | 58 (32) | 22 (26) | 36 (38) | 0.086 |
|  | IV | 12 (7) | 11 (13) | 1 (1) | **0.001** |
| Biology | |  |  |  |  |
|  | Creatinine, µmol/l | 138±61 | 162±74 | 117±33 | **<0.001** |
|  | Glomerular filtration rate, ml/min | 46±19 | 39±18 | 53±18 | **<0.001** |
|  | NT pro-BNP, ng/ml | 4283 [2421-7625] | 4889 [3298-11811] | 3385 [1806-5829] | **<0.001** |
|  | Troponin, ng/ml | 81 [56-126] | 110 [71-153] | 72 [51-101] | **<0.001** |
| Echocardiography | |  |  |  |  |
|  | Left ventricular ejection fraction, % | 48±12 | 45±12 | 51±12 | **0.001** |
|  | Global longitudinal strain, % | 11±4 | 9±4 | 11±4 | **0.001** |
|  | Left atrial volume index, ml/m2 | 56±15 | 56±17 | 56±14 | 0.910 |
|  | Deceleration time, ms | 192±80 | 192±98 | 191±61 | 0.295 |
|  | E/Ea lateral | 15±6 | 16±6 | 15±6 | 0.382 |
| Medications | |  |  |  |  |
|  | Digoxin, n (%) | 3 (2) | 1 (1) | 2 (2) | 0.628 |
|  | Beta-blocker, n (%) | 44 (25) | 30 (35) | 14 (15) | **0.001** |
|  | Amiodarone, n (%) | 93 (52) | 51 (60) | 42 (45) | 0.100 |
|  | Antiplatelet, n (%) | 38 (21) | 24 (28) | 14 (15) | **0.029** |
|  | Switch Anticoagulation | 19 (11) | 8 (9) | 11 (12) | 0.619 |
| Complications | |  |  |  |  |
|  | Sludge or thrombus | 18 (10) | 13 (15) | 5 (5) | 0.800 |
|  | Anticoagulation complication | 26 (15) | 19 (22) | 7 (7) | **0.004** |
|  | Minor bleeding | 12 (7) | 7 (8) | 5 (5) | 0.099 |
|  | Major bleeding | 14 (8) | 12 (14) | 2 (2) | **<0.001** |
|  | Stroke | 8 (4) | 6 (7) | 2 (2) | 0.074 |

DOACs, direct oral anticoagulants; VKAs, vitamin K antagonists

## Demographics of AL population

|  |  | **AL** | **VKAs** | **DOACs** | **P-value** |
| --- | --- | --- | --- | --- | --- |
|  |  | **n= 69** | **n=54** | **n=15** |  |
| Age at diagnosis, years | | 69±10 | 69±10 | 70±12 | 0.760 |
| Male, n (%) | | 40 (58) | 31 (57) | 9 (60) | 0.857 |
| Body mass index, kg/m2 | | 24±4 | 24±4 | 25±2 | 0.775 |
| Diabetes mellitus, n (%) | | 11 (16) | 9 (17) | 2 (13) | 0.755 |
| Vascular disease, n (%) | | 9 (16) | 7 (13) | 2 (13) | 0.990 |
| Hypertension, n (%) | | 27 (39) | 23 (43) | 4 (27) | 0.264 |
| Atrial fibrillation | |  |  |  |  |
|  | Permanent | 28 (41) | 24 (44) | 4 (27) | 0.215 |
|  | CHA2DS2-VASc score | 3±1 | 3±1 | 3±2 | 0.743 |
|  | Emboligene | 11 (16) | 8 (15) | 3 (20) | 0.627 |
| NYHA stage, n (%) | |  |  |  |  |
|  | I | 10 (14) | 8 (15) | 2 (13) | 0.885 |
|  | II | 26 (38) | 18 (33) | 8 (53) | 0.157 |
|  | III | 24 (35) | 20 (37) | 4 (27) | 0.456 |
|  | IV | 9 (13) | 8 (15) | 1 (7) | 0.407 |
| Biology | |  |  |  |  |
|  | Creatinine, µmol/l | 199±159 | 224±171 | 110±32 | **0.002** |
|  | Glomerular filtration rate, ml/min | 42±26 | 38±27 | 57±17 | **0.004** |
|  | NT pro-BNP, ng/ml | 5632 [1844-25991] | 11685 [2628-31058] | 3845 [1365-7827] | 0.220 |
|  | Troponin, ng/ml | 99 [47-198] | 112 [65-210] | 54 [30-149] | 0.118 |
| Echocardiography | |  |  |  |  |
|  | Left ventricular ejection fraction, % | 50±11 | 49±11 | 53±11 | 0.197 |
|  | Global longitudinal strain, % | 11±4 | 11±4 | 11±4 | 0.480 |
|  | Left atrial volume index, ml/m2 | 54±19 | 57±20 | 45±11 | 0.066 |
|  | Deceleration time, ms | 173±50 | 166±50 | 191±46 | 0.172 |
|  | E/Ea lateral | 16±6 | 16±6 | 16±6 | 0.963 |
| Medications | |  |  |  |  |
|  | Digoxin, n (%) | 4 (6) | 4 (7) | 0 (0) | 0.277 |
|  | Beta-blocker, n (%) | 13 (19) | 11 (20) | 2 (13) | 0.538 |
|  | Amiodarone, n (%) | 41 (59) | 32 (59) | 9 (60) | 0.959 |
|  | Antiplatelet, n (%) | 15 (22) | 12 (22) | 3 (20) | 0.854 |
|  | Switch Anticoagulation | 3 (4) | 3 (5) | 0 (0) | 0.351 |
| Complications | |  |  |  |  |
|  | Sludge or thrombus | 7 (10) | 6 (11) | 1 (7) | 0.518 |
|  | Anticoagulation complication | 14 (20) | 12 (22) | 2 (13) | 0.449 |
|  | Minor bleeding | 8 (12) | 6 (11) | 2 (13) | 0.708 |
|  | Major bleeding | 5 (7) | 5 (9) | 0 (0) | 0.092 |
|  | Stroke | 0 (0) | 0(0) | 0 (0) |  |

DOACs, direct oral anticoagulants; VKAs, vitamin K antagonists
